# Supplementary material for: A machine learning contest enhances automated freezing of gait detection and reveals time-of-day effects
Source: Nat Commun. 2024 Jun 6;15:4853. doi: 10.1038/s41467-024-49027-0 (PMC11156937; doi:10.1038/s41467-024-49027-0)
Supplement: Supplementary file 1 — Supplementary Information [file 41467_2024_49027_MOESM1_ESM.pdf]

SUPPLEMENTARY RESULTS

Model Descriptions

5th place (team InnerVoice)

The 5th place winner trained an ensemble of models containing three WaveNet blocks followed by one GRU layer (see Supplementary Figure 1). An ensemble architecture combines the predictions of multiple individual models to increase accuracy and robustness. The autoregressive structure and dilated convolution components of the WaveNet architecture, which shows excellent performances in processing audio data, allow capturing both short-term and long-term dependencies in the data and modeling complex patterns and unique characteristics[1]. Gated recurrent unit (GRU) network architectures are designed with gating mechanisms to process long sequential data, and perform well in natural language processing and speech recognition problems[2-5].

The ensemble was trained in three separate processes: in the first, training was done directly on the fully labeled tDCS FOG and DeFOG data, computing validation score on the continuous validation data irrespective of Valid and Task column values. The two best checkpoints (based on the competition metric, average precision) per fold were selected for the final ensemble. The second training process was similar, but with validation scoring only including valid samples. The third initialized the training with pre-trained weights that were trained on the unlabeled (Daily Living) data before training on the labeled data. The pre-training was performed with the objective of predicting the next element in the time-series.

Acceleration data normalized by a division by a set value were fed into the models, with DeFOG data converted to m/s^2 and tDCS FOG data resampled to 100Hz. Data were segmented into overlapping sequences; short sequences were used for training, while longer sequences were used for inference. Group k-fold cross-validation was implemented with grouping per-subject and a train-validation split of 80-20%. Batches included one DeFOG segment and four tDCS FOG segments, randomly sampled. Binary focal loss was selected as the loss function, treating the problem as multi-label classification. Training was performed over 70-100 epochs, with an Adam optimizer and pytorch OneCycleLR learning rate annealing. Sequence length and patch size were tuned as hyperparameters based on cross-validation and public test scores.

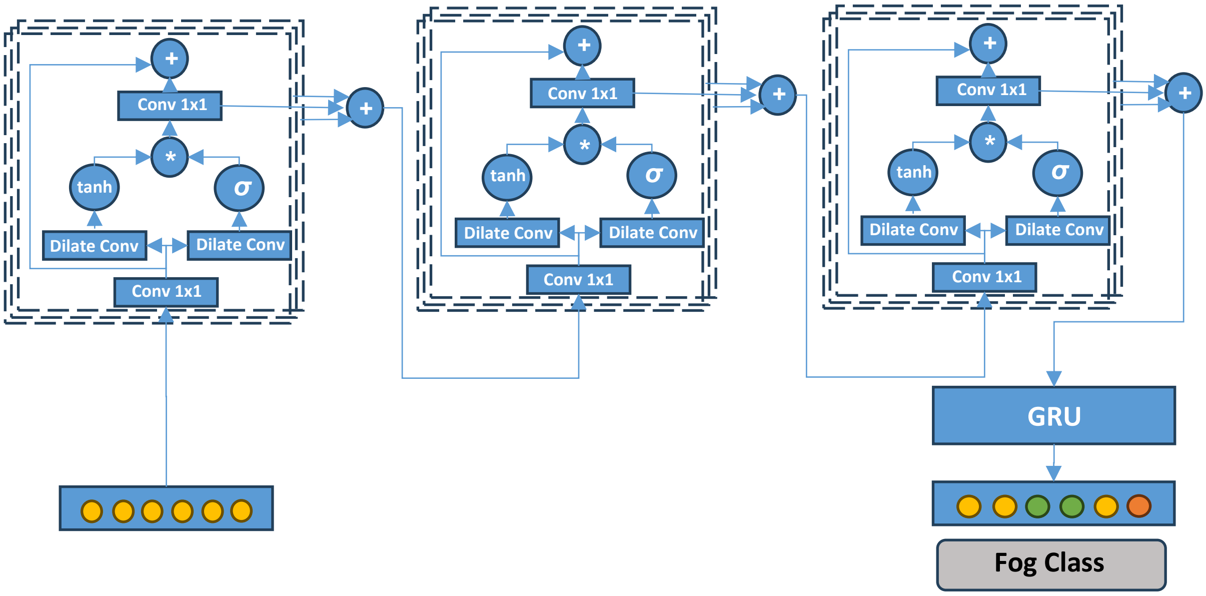

Supplementary Figure 1. 5th place model architecture. Each model in the ensemble comprises three Wavenet blocks containing dilated convolution layers, tanh and sigmoid activations and a residual connection. They are followed by a recurrent GRU layer and finally, a linear fully connected layer which outputs the class probabilities.

4th place (team Zinxira)

The 4th place model used a modified GRU architecture, relying on the following main block: a pure 1-layer bidirectional GRU followed by 2 fully connected layers, with a residual connection adding the input of the block with its output (a schematic representation can be found in Supplementary Figure 2). As commonly done with dense layers in recurrent networks, all the fully connected layers used are time distributed. The dense layer is applied to each time-step of the 1D sequence, i.e., the same weights are used for each time-step – e.g., given a S x 128 1D sequence the input of the dense layer is of size 128 (and not S x 128). After each dense layer a Layer Normalization layer followed by a ReLU activation function is applied.

The bidirectional GRU processes the sequence in the forward and backward directions, to account for past and future contexts[6]. The Residual Bidirectional GRU Block (RBGB) can then be stacked multiple times: in this solution, 3 consecutive blocks are used. Training was performed over 20 epochs with a simple 80-20% validation split, a ranger optimizer and a cosine annealing learning rate schedule starting at the 15th epoch. Mixed precision and gradient clipping[7] were used to speed up the learning and handle the problem of exploding gradients, respectively. A 4th "no-activity" class (where the value is 1 minus the sum of all class confidence scores) was added to the targets and the model was co-trained on both the DeFOG and tDCS FOG class-labeled datasets in an effort to give the model as much global context as possible. As for preprocessing, the sequences were down-sampled to 50Hz. During training, this made processing entire or almost entire sequences with a maximum

length of 200,000 time steps possible at once, along with a batch size of 1, which would not have been possible otherwise given the limitations of the local hardware used. Despite some training instability, it was also empirically observed that this down-sampling significantly helped the learning compared to previous versions of the pipeline implementation. In particular, these experiments led to better performance compared to using gradient accumulation to process entire non-downsampled sequences chunk-by-chunk or using a smaller sequence length. However, it is important to note that the choice of sequence length might not be optimal and studying its impact on the model's performances more thoroughly in future work would be valuable. As for the rest of the preprocessing, DeFOG acceleration units were converted to m/s^2 to match the tDCS FOG data and the data were standardized per-sequence with scikit-learn's StandardScaler, which has proven in practice to be better than global standard normalization with this model. Finally, a mask was applied during the cross-entropy loss computation to include only "Valid" segments of the DeFOG sequences.

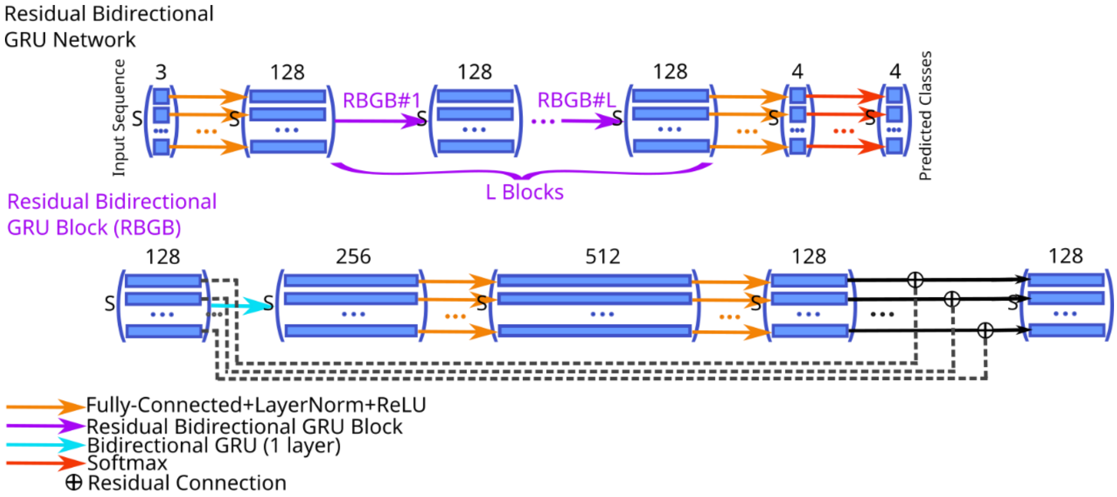

Supplementary Figure 2. 4th place model architecture. Top: the input data are fed to the first RBGB block and propagated through all the blocks of the network until the final fully connected layer, with softmax activation, outputs the class probabilities. Bottom: a schematic representation of a RBGB block in the network.

3rd place (team Stochoshi)

Team Stochoshi primarily used a transformer combined with recurrent neural network architecture in its ensemble. Transformers rely on the concept of "self-attention" to model dependencies among different positions in the input sequence, capturing contextual information. In the transformer architecture, self-attention is applied multiple times in parallel to create "Multi-Head Attention"[8]. Cross-validation was by patient, with stratification by event frequency. All models were trained with focal loss and linear learning rate warmup followed by cosine annealing. Key hyperparameters were 9-15 time points per token and up to 384 tokens.

Most architectures included 2-3 layer transformers - with relative position attention,

disentangled attention, or positional encoding - followed by a GRU layer. Models were trained on a mix of labeled and unlabeled data, with predictions applied to the daily dataset as warmup before final training. The solution handled both labeled datasets as one problem, and relied on heavy augmentations, including stretching, cropping, ablation, and adding Gaussian noise.

2nd place (team Waiwai)

The 2nd place team achieved its results with a GRU ensemble. Other architectures such as LSTM and transformers were considered, but GRU was selected empirically after yielding better results. Handling each of the two labeled datasets separately, the 2nd place solution calculated the cumulative sum (for the tDCS FOG dataset only) and difference between subsequent samples as features, and fed them to the network after normalizing individually per subject. A different normalizing method was used for each dataset: DeFOG data were standardized using scikit-learn's StandardScaler and tDCS FOG data were centered by subtracting the median and scaled by dividing by the interquartile range with scikit-learn's RobustScaler.

The models comprising the tDCS FOG ensemble differed in the class weighting scheme used to calculate the binary cross-entropy with logits loss. Those in the DeFOG ensemble varied in their GRU hidden layer size, the amount of data used in training, and all but one of them utilized 'notype' data, by creating pseudo-labels iteratively over two iterations using models trained first on fully labeled DeFOG data and then on both the labeled (DeFOG) and pseudo-labeled (notype) data. For training an AdamW optimizer and a linear learning rate warmup and decay schedule were implemented, along with a stratified group k-fold cross-validation that grouped samples by subject. Similarly to the 5th place solution, training was done on short overlapping sequences and inference used longer ones.

1st place (team Baurzhan Urazalinov)

The top-ranking model was an ensemble of transformer encoders with bidirectional long short-term memory (BiLSTM) layers (see Supplementary Figure 3). The BiLSTM layers allows for the modeling of long-term dependencies in a data sequence, while also capturing bidirectional contexts[6]. In the winning solution, the data were processed as two-dimensional patches rather than sequences, similar to a visual transformer[9], with the second dimension being the three acceleration axes. Further processing included standardization per-signal and a reduction of the target resolution. Two different models were implemented to optimize the results on the two labeled datasets, using a patch size of 18 and 14 for the tDCS FOG and the DeFOG model, respectively. Data were split by subject for training, with an 85-15% split for training and validation. The training was done with an Adam optimizer, a binary cross-entropy loss function, and a linear learning rate warm-up schedule up to a selected learning rate value.

Instead of manually creating features from acceleration values and using short windows, the winner chose to organize sequences of acceleration into a long sequence of patches. With sufficient depth of the neural network, the use of patches allows the model to automatically

generate useful features from acceleration values during training. He selected the patch size individually for each dataset during training based on validation scores. The length of the patch sequence was selected similarly. However, this parameter may not be as important compared to other model parameters, such as patch size and the number of encoder layers and BiLSTM layers. The model was effective with a patch sequence length of 864, which means that the model can handle long segments. This is a good indication that at each local location, the model richly utilizes information from the entire segment, which, presumably, should enhance the capabilities of the deep neural network to localize FOG episodes.

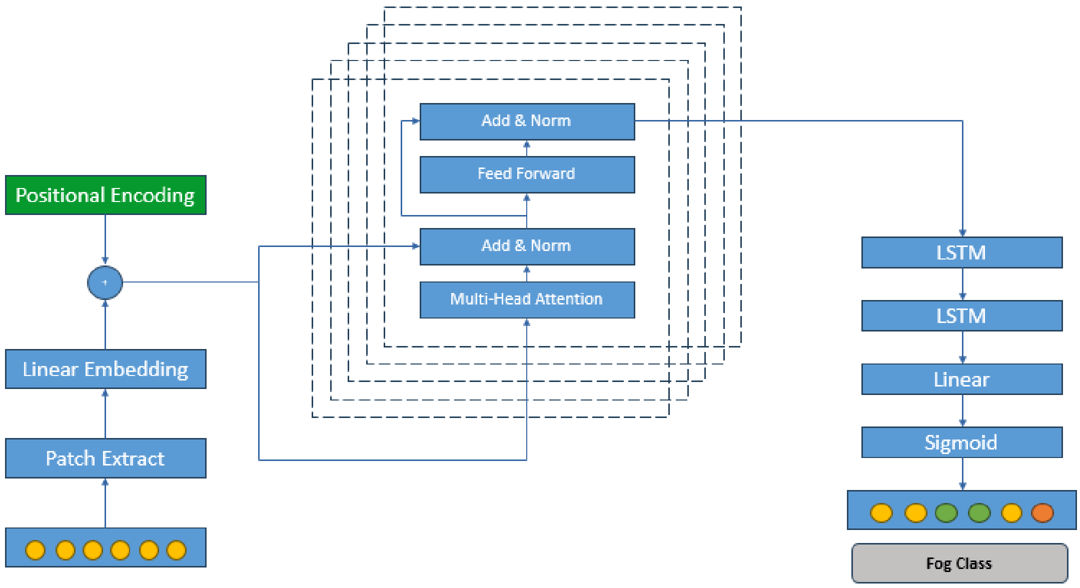

Supplementary Figure 3. 1st place model architecture. Each model in the ensemble comprises a transformer-encoder part and a recurrent part. Inputs are embedded and positional encoding is applied. Then the information is fed through the attention and feed-forward layers, with normalization and residual connections as illustrated. Finally, it propagates through two BiLSTM layers, a linear layer, and sigmoid activation to produce the class probabilities.

Evaluation metrics on private+public test data

The evaluation metrics were also computed for the full test dataset. They are presented in Supplementary Table 1.

Supplementary Table 1: Evaluation metrics of the top 5 models on the private and public test data (n=40) at the point on the precision-recall curve closest to (1,1).						
Team	FOG class	F1 score	Accuracy	Precision	Recall	Specificity
1st place	Start Hesitation	0.479	0.995	0.766	0.348	0.999
	Turn	0.758	0.871	0.715	0.806	0.893
	Walk	0.243	0.808	0.143	0.811	0.808
	All FOG	0.777	0.860	0.733	0.826	0.874
2nd place	Start Hesitation	0.370	0.992	0.424	0.328	0.997
	Turn	0.772	0.882	0.745	0.801	0.909
	Walk	0.535	0.960	0.479	0.605	0.974
	All FOG	0.800	0.878	0.772	0.831	0.897
3rd place	Start Hesitation	0.345	0.984	0.246	0.579	0.987
	Turn	0.791	0.890	0.751	0.836	0.908
	Walk	0.385	0.923	0.276	0.634	0.934
	All FOG	0.768	0.855	0.726	0.815	0.871
4th place	Start Hesitation	0.499	0.993	0.496	0.503	0.996
	Turn	0.740	0.862	0.697	0.787	0.886
	Walk	0.146	0.625	0.080	0.843	0.616
	All FOG	0.761	0.852	0.724	0.803	0.872
5th place	Start Hesitation	0.639	0.995	0.694	0.592	0.998
	Turn	0.761	0.874	0.772	0.804	0.897
	Walk	0.147	0.634	0.081	0.827	0.627
	All FOG	0.755	0.852	0.740	0.771	0.887

Significance of correlations between model predictions and gold-standard measures

Supplementary Table 2: P-values of intra class correlation results reflecting the model's ability to reproduce gold-standard measures based on expert review of the videos (see also Table 2).						
		1st place	2nd place	3rd place	4th place	5th place
% Time Frozen	Private test	$2.21e^{-8}$	$7.10e^{-8}$	$1.05e^{-7}$	$7.25e^{-6}$	$1.11e^{-5}$
	Private + public test	$1.26e^{13}$	$2.42e^{-15}$	$8.83e^{-16}$	$8.24e^{-14}$	$6.02e^{-13}$
No. of FOG episodes	Private test	$8.02e^{-6}$	$1.91e^{-5}$	0.001	0.311	$8.40e^{-6}$
	Private + public test	$6.16e^{-5}$	0.001	$1.99e^{-6}$	0.211	0.014
FOG Duration	Private test	$8.70e^{-13}$	$5.47e^{-13}$	$2.13e^{-11}$	$4.44e^{-9}$	$1.81e^{-11}$
	Private + public test	$3.04e^{-22}$	$2.17e^{-21}$	$9.80e^{-25}$	$1.85e^{-21}$	$2.77e^{-17}$

P-values are the results ICC(2, 1) test.

Actual (based on the review of the videos by experts) and model estimated values of FOG measures

Supplementary Table 3: Measures of FOG occurrences quantified in the two test sets and estimated by the winning models (median [IQR]).				
Private test: n=14<br>Private + Public test: n=40		% Time Frozen	No. of FOG episodes	FOG Duration [s]
Gold standard, video-based	Private test	9.15 [3.76-18.81]	25.50 [15.25-30.75]	58.33 [26.65-154.02]
	Private + public test	14.96 [5.78-33.87]	27.00 [14.75-44.25]	98.32 [40.31-352.36]
1st place	Private test	7.57 [0.18-16.97]	9.00 [1.25-24.25]	50.04 [1.90-180.62]
	Private + public test	16.14 [2.42-34.3]	13.50 [7.25-29.00]	119.58 [14.41-399.73]
2nd place	Private test	2.16 [0.01-16.38]	23.00 [0.25-39.50]	16.85 [0.07-139.67]
	Private + public test	10.88 [1.14-38.33]	31.00 [9.75-53.25]	71.60 [10.17-358.10]
3rd place	Private test	5.46 [1.16-22.41]	21.00 [8.25-48.50]	35.68 [5.77-151.81]
	Private + public test	13.06 [3.80-42.87]	38.00 [18.00-70.25]	102.64 [31.45-419.37]
4th place	Private test	4.56 [2.86-15.50]	91.00 [32.00-167.25]	36.44 [16.38-125.65]
	Private + public test	16.57 [3.15-39.45]	120.50 [53.50-181.00]	116.44 [31.49-418.44]
5th place	Private test	11.83 [9.03-17.84]	20.50 [10.25-35.00]	87.72 [12.75-135.14]
	Private + public test	13.04 [6.05-27.72]	31.50 [14.00-46.75]	97.95 [35.81-314.31]

Post-competition analyses without overlap of subjects between test and training datasets

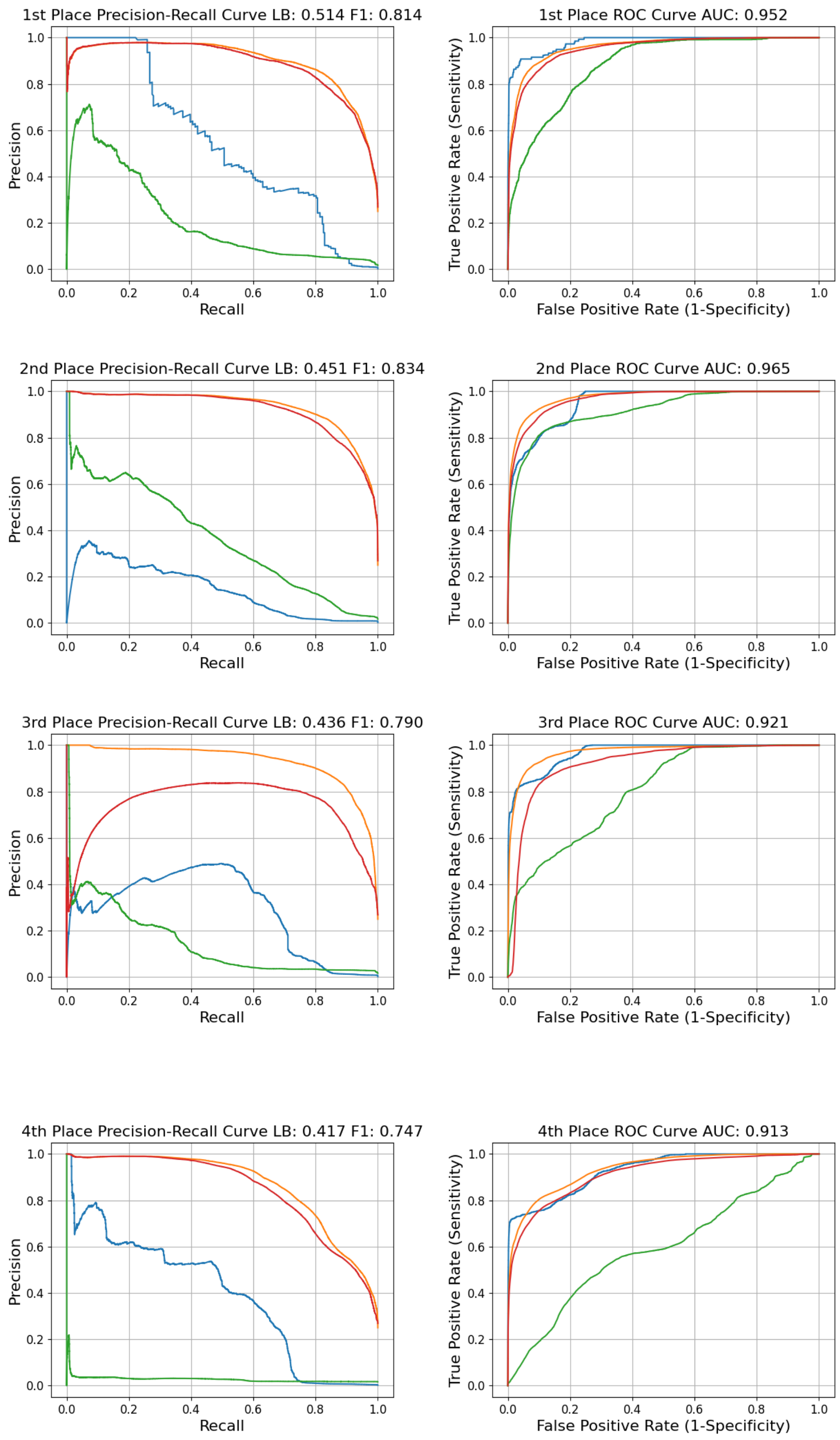

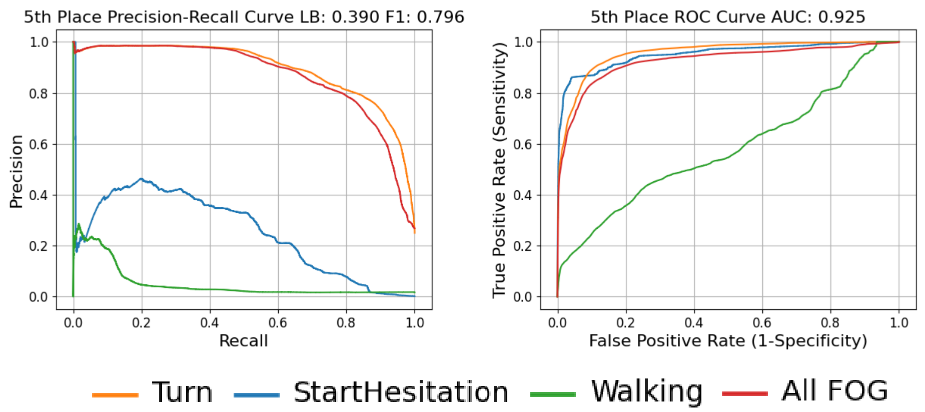

Supplementary Figure 4. Precision-recall curves (left) and receiver operating characteristic (ROC) curves (right) for the top five ranked models based on the private (hidden) test set, after excluding subjects overlapping with train data. Curve colors indicate the FOG class, as seen in the figure legend. When comparing the results with and without the inclusion of the overlapping subjects, the % of change of the area under the ROC curve was smaller than 1% for all curves.

Supplementary Table 4: Evaluation metrics of all FOG classes of the top 5 models on the private (hidden) test data at the point on the precision-recall curve closest to (1,1) after excluding subjects overlapping with train data.						
Team	FOG class	F1 score	Accuracy	Precision	Recall	Specificity
1st place	Start Hesitation	0.515	0.998	0.524	0.506	0.999
	Turn	0.835	0.918	0.840	0.831	0.947
	Walk	0.085	0.665	0.045	0.926	0.661
	All FOG	0.814	0.900	0.817	0.811	0.933
2nd place	Start Hesitation	0.196	0.990	0.118	0.573	0.991
	Turn	0.859	0.929	0.855	0.863	0.951
	Walk	0.426	0.980	0.417	0.435	0.990
	All FOG	0.834	0.912	0.848	0.821	0.946
3rd place	Start Hesitation	0.504	0.998	0.475	0.537	0.999
	Turn	0.861	0.930	0.853	0.868	0.950
	Walk	0.056	0.458	0.029	0.960	0.450
	All FOG	0.790	0.884	0.768	0.814	0.909
4th place	Start Hesitation	0.498	0.998	0.537	0.465	0.999
	Turn	0.772	0.887	0.781	0.763	0.929
	Walk	0.034	0.064	0.017	0.985	0.048
	All FOG	0.747	0.866	0.757	0.737	0.913
5th place	Start Hesitation	0.400	0.997	0.328	0.511	0.998
	Turn	0.817	0.905	0.790	0.845	0.925
	Walk	0.035	0.080	0.018	1.000	0.065
	All FOG	0.796	0.887	0.776	0.817	0.913

Supplementary Table 5: % of change of evaluation metrics after excluding subjects overlapping with train data, compared to before excluding these subjects.						
Team	FOG class	F1 score	Accuracy	Precision	Recall	Specificity
1st place	Start Hesitation	12.2	0.2	51.4	-25.7	0.2
	Turn	2.6	-0.2	2.3	3.0	-0.4
	Walk	7.6	-2.5	9.8	1.2	-2.5
	All FOG	3.0	-0.4	1.5	4.6	-1.1
2nd place	Start Hesitation	1300.0	46.9	1585.7	-40.3	47.3
	Turn	2.5	-0.2	0.2	4.9	-1.0
	Walk	4.7	-0.3	0.0	9.0	-0.2
	All FOG	2.7	-0.3	1.1	4.5	-0.8
3rd place	Start Hesitation	14.8	0.1	6.7	23.7	0.0
	Turn	2.1	-0.3	-0.2	4.3	-1.1
	Walk	5.7	-3.8	7.4	-1.2	-4.1
	All FOG	3.7	-0.1	4.1	3.3	-0.7
4th place	Start Hesitation	0.6	0.0	-7.9	8.1	0.0
	Turn	2.4	-0.9	2.6	2.1	-0.6
	Walk	6.3	-44.8	6.3	0.5	-53.4
	All FOG	2.5	-1.1	2.0	2.9	-1.2
5th place	Start Hesitation	7.2	-0.1	-27.9	61.2	-0.1
	Turn	3.5	0.0	3.9	3.0	-0.4
	Walk	12.9	12.7	12.5	0.0	14.0
	All FOG	3.9	-0.3	2.1	6.0	-1.4

*Positive values indicate better performance after excluding the subjects. Negative values indicate worse performance after excluding the subjects. For the 1st – 5th place model, Turn and ALL FOG results differed (with versus without overlap) by less than 5% for almost all of the measures.

Supplementary Table 6A: Intraclass correlation results reflecting the model's ability to reproduce gold-standard measures based on expert review of the videos after excluding subjects overlapping with train data.

ICCs (CI: 95%)		1st place	2nd place	3rd place	4th place	5th place
% Time Frozen	Private test	0.947** (0.80-0.99)	0.949** (0.80-0.99)	0.949** (0.79-0.99)	0.874* (0.53-0.97)	0.878* (0.55-0.97)
	Private + public test	0.840** (0.68-0.92)	0.882** (0.76-0.94)	0.902** (0.80-0.95)	0.847** (0.70-0.93)	0.829** (0.66-0.92)
No. of FOG episodes	Private test	0.697** (-0.07-0.93)	0.832* (0.45-0.96)	0.651* (0.09-0.91)	0.010 (-0.21-0.45)	0.845* (0.44-0.96)
	Private + public test	0.399* (0.05-0.66)	0.362* (-0.01-0.64)	0.558** (0.22-0.77)	0.060 (-0.14-0.32)	0.213 (-0.17-0.54)
FOG Duration	Private test	0.990** (0.96-1.00)	0.992** (0.96-1.00)	0.984** (0.93-1.00)	0.963** (0.85-0.99)	0.984** (0.93-1.00)
	Private + public test	0.946** (0.89-0.97)	0.940** (0.87-0.97)	0.971** (0.94-0.99)	0.939** (0.87-0.97)	0.884** (0.74-0.95)

Supplementary Table 6B: P-values of intra class correlation results reflecting the model's ability to reproduce gold-standard measures based on expert review of the videos after excluding subjects overlapping with train data.

ICCs (CI: 95%)		1st place	2nd place	3rd place	4th place	5th place
% Time Frozen	Private test	$1.68e^{-5}$	$1.92e^{-5}$	$2.13e^{-5}$	$6.87e^{-4}$	$6.09e^{-4}$
	Private + public test	$8.92e^{-9}$	$1.26e^{-10}$	$1.13e^{-11}$	$3.13e^{-9}$	$8.05e^{-9}$
No. of FOG episodes	Private test	$7.46e^{-4}$	0.002	0.018	0.476	0.002
	Private + public test	0.007	0.028	$2.57e^{-4}$	0.300	0.138
FOG Duration	Private test	$3.10e^{-8}$	$1.51e^{-8}$	$1.85e^{-7}$	$5.93e^{-6}$	$2.01e^{-7}$
	Private + public test	$7.03e^{-15}$	$1.02e^{-14}$	$9.50e^{-19}$	$3.41e^{-14}$	$1.83e^{-11}$

*p<0.05, **p<0.001 based on an ICC(2,1) test.

Supplementary Table 7: % of change of intraclass correlation after excluding subjects overlapping with train data, compared to without the exclusion of these subjects						
ICCs (CI: 95%)		1st place	2nd place	3rd place	4th place	5th place
% Time Frozen	Private test	-0.2	1.6	0.7	-1.4	0.1
	Private + public test	-3.3	-0.2	0.4	-2.6	-2.7
No. of FOG episodes	Private test	-8.7	-4.3	-9.2	-89.2	-4.5
	Private + public test	-20.2	-20.6	-6.5	-28.6	-38.4
FOG Duration	Private test	-0.1	0.1	-0.1	-0.2	-0.1
	Private + public test	-0.9	-0.4	0.6	-1.2	-2.5

* Positive values indicate better performance after excluding the subjects. Negative values indicate worse performance after excluding the subjects. For % time frozen and FOG duration, the differences between with and without overlap were less than 4% for all 5 top models for the private and private+public test sets.

Supplementary Table 8: Measures of FOG occurrences quantified in the two test sets and estimated by the winning models (mean±SD), after excluding subjects overlapping with train data

Private test: n=9<br>Private + Public test: n=28		% Time Frozen	No. of FOG episodes	FOG Duration [s]
Gold standard, video-based	Private test	18.83±17.41	37.67±21.86	270.20±423.30
	Private + public test	23.98±21.90	38.00±25.50	307.08±361.99
1st place	Private test	17.16±19.57	22.89±20.15	259.45±452.52
	Private + public test	24.62±23.41	24.50±23.25	292.51±358.85
2nd place	Private test	17.85±20.10	42.33±30.27	266.96±443.23
	Private + public test	22.01±22.84	43.39±40.14	270.95±360.22
3rd place	Private test	18.41±20.90	51.78±51.92	283.89±485.74
	Private + public test	25.90±24.38	52.89±40.07	325.75±400.43
4th place	Private test	19.12±20.40	192.33±135.16	264.92±475.77
	Private + public test	26.74±24.37	205.32±192.58	321.70±396.19
5th place	Private test	19.61±19.17	37.44±28.42	268.54±444.18
	Private + public test	20.26±21.67	43.29±42.02	237.29±335.02

Inference Runtime

Supplementary Table 9 shows the run times of the top 5 models during submission (i.e., when applying their final models to the test data) on the Kaggle platform.

Supplementary Table 9: Final submission (inference on test) run times in seconds

	Time	Device
1st place	2,301	P100 GPU
2nd place	2,984	P100 GPU
3rd place	769	P100 GPU
4th place	831	P100 GPU
5th place	10,488	CPU

More information on the Kaggle hardware technical specifications is available on the Kaggle website: https://www.kaggle.com/docs/notebooks#technical-specifications

REFERENCES

1 van den Oord, A. *et al.* WaveNet: A Generative Model for Raw Audio. arXiv:1609.03499 (2016). <https://ui.adsabs.harvard.edu/abs/2016arXiv160903499V>.

2 Chung, J., Gulcehre, C., Cho, K. & Bengio, Y. Empirical Evaluation of Gated Recurrent Neural Networks on Sequence Modeling. arXiv:1412.3555 (2014). <https://ui.adsabs.harvard.edu/abs/2014arXiv1412.3555C.>

3 Yin, W., Kann, K., Yu, M. & Schütze, H. Comparative Study of CNN and RNN for Natural Language Processing. arXiv:1702.01923 (2017). <https://ui.adsabs.harvard.edu/abs/2017arXiv170201923Y>.

4 Kumar, A. *et al.* in *Proceedings of The 33rd International Conference on Machine Learning* Vol. 48 (eds Balcan Maria Florina & Q. Weinberger Kilian) 1378--1387 (PMLR, Proceedings of Machine Learning Research, 2016).

5 Shewalkar, A., Nyavanandi, D. & Ludwig, S. A. Performance Evaluation of Deep Neural Networks Applied to Speech Recognition: RNN, LSTM and GRU. *Journal of Artificial Intelligence and Soft Computing Research* **9**, 235-245 (2019). https://doi.org:doi:10.2478/jaiscr-2019-0006

6 Graves, A. & Schmidhuber, J. Framewise phoneme classification with bidirectional LSTM and other neural network architectures. *Neural Networks* **18**, 602-610 (2005). https://doi.org:https://doi.org/10.1016/j.neunet.2005.06.042

7 Pascanu, R ,.Mikolov, T. & Bengio, Y. in *Proceedings of the 30th International Conference on Machine Learning* Vol. 28 (eds Dasgupta Sanjoy & McAllester David) 1310--1318 (PMLR, Proceedings of Machine Learning Research, 2013).

8 Vaswani, A. *et al.* Attention is all you need. *Advances in neural information processing systems* **30** .(2017)

9 Dosovitskiy, A. *et al.* An Image is Worth 16x16 Words: Transformers for Image Recognition at Scale. arXiv:2010.11929 (2020). <https://ui.adsabs.harvard.edu/abs/2020arXiv201011929D.>
