# Supplementary material for: A machine learning contest enhances automated freezing of gait detection and reveals time-of-day effects
Source: Nat Commun. 2024 Jun 6;15:4853. doi: 10.1038/s41467-024-49027-0 (PMC11156937; doi:10.1038/s41467-024-49027-0)
Supplement: Supplementary file 2 — Reporting Summary [file 41467_2024_49027_MOESM2_ESM.pdf]

nature portfolio

Corresponding author(s): Jeffrey Hausdorff

Last updated by author(s): Apr 11, 2024

Reporting Summary

Nature Portfolio wishes to improve the reproducibility of the work that we publish. This form provides structure for consistency and transparency in reporting. For further information on Nature Portfolio policies, see our Editorial Policies and the Editorial Policy Checklist.

Statistics

For all statistical analyses, confirm that the following items are present in the figure legend, table legend, main text, or Methods section.

n/a	Confirmed	
☐	☒	The exact sample size (*n*) for each experimental group/condition, given as a discrete number and unit of measurement
☐	☒	A statement on whether measurements were taken from distinct samples or whether the same sample was measured repeatedly
☐	☒	The statistical test(s) used AND whether they are one- or two-sided<br>*Only common tests should be described solely by name; describe more complex techniques in the Methods section.*
☒	☐	A description of all covariates tested
☐	☒	A description of any assumptions or corrections, such as tests of normality and adjustment for multiple comparisons
☐	☒	A full description of the statistical parameters including central tendency (e.g. means) or other basic estimates (e.g. regression coefficient) AND variation (e.g. standard deviation) or associated estimates of uncertainty (e.g. confidence intervals)
☐	☒	For null hypothesis testing, the test statistic (e.g. *F*, *t*, *r*) with confidence intervals, effect sizes, degrees of freedom and *P* value noted<br>*Give P values as exact values whenever suitable.*
☒	☐	For Bayesian analysis, information on the choice of priors and Markov chain Monte Carlo settings
☒	☐	For hierarchical and complex designs, identification of the appropriate level for tests and full reporting of outcomes
☒	☐	Estimates of effect sizes (e.g. Cohen's *d*, Pearson's *r*), indicating how they were calculated

Our web collection on statistics for biologists contains articles on many of the points above.

Software and code

Policy information about availability of computer code

Data collection	Data collection was not in the scope of this manuscript. The competition used existing datasets. The existing data were organized using MATLAB R2021b
Data analysis	Python 3.9, with packages: Numpy 1.21.5, Pandas 1.5.3, Pingouin 0.5.3, Scikit-learn 1.2.2<br>MATLAB R2021b, SPSS 29.0.0.0, GraphPad Prism 10.0.2<br>Custom code repository link: https://doi.org/10.5281/zenodo.10653029

For manuscripts utilizing custom algorithms or software that are central to the research but not yet described in published literature, software must be made available to editors and reviewers. We strongly encourage code deposition in a community repository (e.g. GitHub). See the Nature Portfolio guidelines for submitting code & software for further information.

Data

Policy information about availability of data

All manuscripts must include a data availability statement. This statement should provide the following information, where applicable:

- Accession codes, unique identifiers, or web links for publicly available datasets
- A description of any restrictions on data availability
- For clinical datasets or third party data, please ensure that the statement adheres to our policy

The data used in this work are available at the competition webpage: https://www.kaggle.com/competitions/tlvmc-parkinsons-freezing-gait-prediction/data

A copy that includes the test data is available at: https://doi.org/10.5281/zenodo.10959560

The figure data generated in this study are provided in the Source Data files at the same link.

Research involving human participants, their data, or biological material

Policy information about studies with human participants or human data. See also policy information about sex, gender (identity/presentation), and sexual orientation and race, ethnicity and racism.

Reporting on sex and gender	Since research to date has not reported any association between the behavioral manifestation of FOG and sex or gender, no sex or gender analysis was carried out. Participant sex is reported based on self-report. Disaggregated sex or gender data were not collected. Overall, 41 females and 106 males were included in this study.
Reporting on race, ethnicity, or other socially relevant groupings	Socially constructed or relevant categorization variables were not considered in this analysis. It is not known, based on existing literature, that such variables are related to FOG. This information was not collected in the original studies in which the data were collected.
Population characteristics	No covariant-relevant population characteristics were analyzed. We describe age, disease severity, and duration (based on self-report and clinical evaluation) in table 6 of the manuscript.
Recruitment	Patients were identified from local clinics, existing databases, and referral from movement disorders specialists. Subjects were all studied at academic research centers and may, therefore, not fully represent the general population of patients with Parkinson's disease and freezing of gait. Nonetheless, there was a relatively large range in disease and FOG-severity.
Ethics oversight	The original studies received human studies approvals by the local human studies committee at Hebrew SeniorLife (IRB-2016-13), the Tel Aviv Sourasky Medical Center (0710-15-TLV, 0674-15-TLV, 0908-18-TLV) and UZ/KU Leuven (internal reference number: s62453). Approvals were obtained to use the de-identified data for this machine learning contest.

Note that full information on the approval of the study protocol must also be provided in the manuscript.

Field-specific reporting

Please select the one below that is the best fit for your research. If you are not sure, read the appropriate sections before making your selection.

[x] Life sciences [ ] Behavioural & social sciences [ ] Ecological, evolutionary & environmental sciences

For a reference copy of the document with all sections, see nature.com/documents/nr-reporting-summary-flat.pdf

Life sciences study design

All studies must disclose on these points even when the disclosure is negative.

Sample size	No formal sample size calculation was conducted for this machine learning context. We utilized available datasets. We note that the sample size was generally much larger than previous reports in the literature, suggesting that it would be sufficient.
Data exclusions	All available usable data were included in the analysis.
Replication	Multiple steps have been made to enable replication. For example, all of the models and the data are open access.
Randomization	Dataset splits into train and test sets were randomized by Kaggle according to Kaggle protocols.
Blinding	The analyses of the test results were done in a blinded manner, with automatic code that produced the performance metrics.

Reporting for specific materials, systems and methods

We require information from authors about some types of materials, experimental systems and methods used in many studies. Here, indicate whether each material, system or method listed is relevant to your study. If you are not sure if a list item applies to your research, read the appropriate section before selecting a response.

Materials & experimental systems

n/a	Involved in the study
☒	☐ Antibodies
☒	☐ Eukaryotic cell lines
☒	☐ Palaeontology and archaeology
☒	☐ Animals and other organisms
☐	☒ Clinical data
☒	☐ Dual use research of concern
☒	☐ Plants

Methods

n/a	Involved in the study
☒	☐ ChIP-seq
☒	☐ Flow cytometry
☒	☐ MRI-based neuroimaging

Clinical data

Policy information about clinical studies
All manuscripts should comply with the ICMJEguidelines for publication of clinical research and a completedCONSORT checklist must be included with all submissions.

Clinical trial registration	NCT02656316 (tDCS FOG), NCT03978507 (DeFOG)
Study protocol	The study protocols for each of the included datasets are described in their original manuscripts, as cited in Methods.
Data collection	tDCS FOG patients included in the competition were recruited in Boston and Tel Aviv between September 2016 and September 2019 and their data was collected from September 2016 to November 2019.<br>DeFOG patients that were included in the competition (baseline) were recruited in Tel Aviv and Leuven from June 2019 to June 2022 and their data were collected during that period.<br>ONPar patients were recruited in Tel Aviv from July 2017 to March 2021. The data were collected from October 2017 to March 2021.
Outcomes	The three FOG outcomes (% time frozen, number of FOG events, total FOG duration) are pre-defined outcomes of FOG based on the existing literature.

Plants

Seed stocks	*Report on the source of all seed stocks or other plant material used. If applicable, state the seed stock centre and catalogue number. If plant specimens were collected from the field, describe the collection location, date and sampling procedures.*
Novel plant genotypes	*Describe the methods by which all novel plant genotypes were produced. This includes those generated by transgenic approaches, gene editing, chemical/radiation-based mutagenesis and hybridization. For transgenic lines, describe the transformation method, the number of independent lines analyzed and the generation upon which experiments were performed. For gene-edited lines, describe the editor used, the endogenous sequence targeted for editing, the targeting guide RNA sequence (if applicable) and how the editor was applied.*
Authentication	*Describe any authentication procedures for each seed stock used or novel genotype generated. Describe any experiments used to assess the effect of a mutation and, where applicable, how potential secondary effects (e.g. second site T-DNA insertions, mosiacism, off-target gene editing) were examined.*
